# BCG Vaccination Reduces Risk of Tuberculosis Infection in Vaccinated Badgers and Unvaccinated Badger Cubs

**DOI:** 10.1371/journal.pone.0049833

**Published:** 2012-12-12

**Authors:** Stephen P. Carter, Mark A. Chambers, Stephen P. Rushton, Mark D. F. Shirley, Pia Schuchert, Stéphane Pietravalle, Alistair Murray, Fiona Rogers, George Gettinby, Graham C. Smith, Richard J. Delahay, R. Glyn Hewinson, Robbie A. McDonald

**Affiliations:** 1 The Food and Environment Research Agency, York, North Yorkshire, United Kingdom; 2 Department of Bacteriology, Animal Health and Veterinary Laboratories Agency, Addlestone, Surrey, United Kingdom; 3 School of Biology, University of Newcastle, Newcastle upon Tyne, United Kingdom; 4 Department of Mathematics and Statistics, University of Strathclyde, Glasgow, United Kingdom; 5 Environment and Sustainability Institute, University of Exeter, Penryn, Cornwall, United Kingdom; Hopital Raymond Poincare - Universite Versailles St. Quentin, France

## Abstract

Wildlife is a global source of endemic and emerging infectious diseases. The control of tuberculosis (TB) in cattle in Britain and Ireland is hindered by persistent infection in wild badgers (*Meles meles*). Vaccination with Bacillus Calmette-Guérin (BCG) has been shown to reduce the severity and progression of experimentally induced TB in captive badgers. Analysis of data from a four-year clinical field study, conducted at the social group level, suggested a similar, direct protective effect of BCG in a wild badger population. Here we present new evidence from the same study identifying both a direct beneficial effect of vaccination in individual badgers and an indirect protective effect in unvaccinated cubs. We show that intramuscular injection of BCG reduced by 76% (Odds ratio = 0.24, 95% confidence interval (CI) 0.11–0.52) the risk of free-living vaccinated individuals testing positive to a diagnostic test combination to detect progressive infection. A more sensitive panel of tests for the detection of infection *per se* identified a reduction of 54% (Odds ratio = 0.46, 95% CI 0.26–0.88) in the risk of a positive result following vaccination. In addition, we show the risk of unvaccinated badger cubs, but not adults, testing positive to an even more sensitive panel of diagnostic tests decreased significantly as the proportion of vaccinated individuals in their social group increased (Odds ratio = 0.08, 95% CI 0.01–0.76; *P* = 0.03). When more than a third of their social group had been vaccinated, the risk to unvaccinated cubs was reduced by 79% (Odds ratio = 0.21, 95% CI 0.05–0.81; *P* = 0.02).

## Introduction

There is growing recognition of the importance of wildlife hosts in the emergence, spread and maintenance of infectious diseases that severely compromise human and animal health, inflict major economic costs and threaten biodiversity [1], [2]. Bovine tuberculosis (TB), caused by infection with *Mycobacterium bovis*, is a globally important animal disease and the most serious health threat to the cattle industry in Britain and Ireland. Over 10% of herds in England alone were under disease control restrictions at some point between 2010 and 2011 as a result of incidents of TB, resulting in the compulsory slaughter of nearly 25,000 cattle [3]. The associated annual costs including testing, research and compensation exceeded £90M [3].

The control of TB in cattle in Britain and Ireland has been seriously constrained by the presence of a persistent reservoir of infection in Eurasian badgers (*Meles meles*) [4]. There is also growing evidence implicating badgers in the persistence of *M. bovis* infection in parts of mainland Europe, although other wildlife species currently appear to be more important maintenance hosts outside of the British Isles [5]. Badger culling has been employed in Britain and Ireland in an attempt to reduce TB in cattle. Localised culling trials in the Republic of Ireland (RoI) have reported some considerable success in reducing incidence of TB in cattle in these areas [6], [7], [8], and badgers are now generally culled in response to local cattle herd breakdowns (CHBs) in the ROI [9]. However, in Britain where the density of badgers is generally higher and the wider effects of culling have been investigated, badger culling has resulted in both increases and decreases in cattle TB [10], [11], [12], [13], [14], [15]. Also, badgers are a protected and iconic species in Britain and culling badgers remains highly controversial [16], [17], [18].

Vaccination has been used successfully to manage wildlife reservoirs of infection, but has largely been limited to the control of acute viral diseases, such as rabies in Europe and North America [19], [20] and Classical Swine Fever, where the vaccination of wild boar has achieved some success [21], [22]. So far, vaccination has not been used extensively to control chronic bacterial infections, such as TB in wildlife [23].

Previous work with captive badgers has demonstrated that the intramuscular administration of Bacillus Calmette-Guérin (BCG) confers a degree of direct protection in vaccinated individuals. In experimental challenge studies, BCG vaccination significantly reduced disease progression and severity, and excretion of *M. bovis* infection in individual badgers [24], [25]. Initial results from a parallel field study showed that vaccination significantly reduced the incidence of positive responses to a serological test for TB (Stat-Pak, Chembio Diagnostic Systems Inc., New York, USA) suggesting a beneficial effect of BCG in reducing disease progression in wild badgers [24]. That study was undertaken as part of a blinded-trial where the social group was the designated treatment unit and the initial analysis did not consider the effects of vaccination at the level of the individual badger, consequently factors such as the time individuals entered and exited the study (e.g. through recruitment, dispersal and mortality) were not accounted for.

A specific challenge associated with prophylactic vaccination arises if a high proportion of individuals cannot be vaccinated before they become infected [26]. This is a particular concern for the vaccination of badgers against TB because they live in close contact with one another and their young do not generally emerge from their underground den (sett) for the first two months of their life [27]. The argument that many badger cubs will become infected during this period (i.e. before they can be caught and vaccinated), has been identified as a key potential constraint on the effectiveness of badger vaccination as a management tool [28]. Under such circumstances, indirect effects of vaccination may be important. The concept of non-immunised individuals within a group or population being ‘protected’ from disease transmission by the presence and proximity of immune individuals is long established [29]. The term “herd immunity” was subsequently coined to describe this phenomenon [30] and has since become an integral component of the science underpinning human vaccination programmes [31]. Herd immunity underlies the global eradication of smallpox, though evidence for it in human TB vaccination campaigns is limited [32].

In this paper we report further analyses of results from the four-year clinical field study of the effects of parenteral administration of BCG in wild badgers. We extend the previous study of the effects of BCG on wild badgers by investigating the direct effect of vaccination on individual badgers and the indirect effects on unvaccinated badgers. Neither approach was possible previously due to blinding of the data. Data were initially blinded as they constituted safety and supporting efficacy evidence for a marketing authorisation for parenterally administered BCG in badgers, which was subsequently granted in March 2010.

## Methods

### Ethics statement

All animal procedures were covered by licences issued by the Home Office and the Veterinary Medicines Directorate, following approval by ethics panels at The Food and Environment Research Agency and Animal Health and Veterinary Laboratories Agency. The study was conducted according to the principles of Good Clinical Practice [33].

### Study area and population

**Figure 1:**
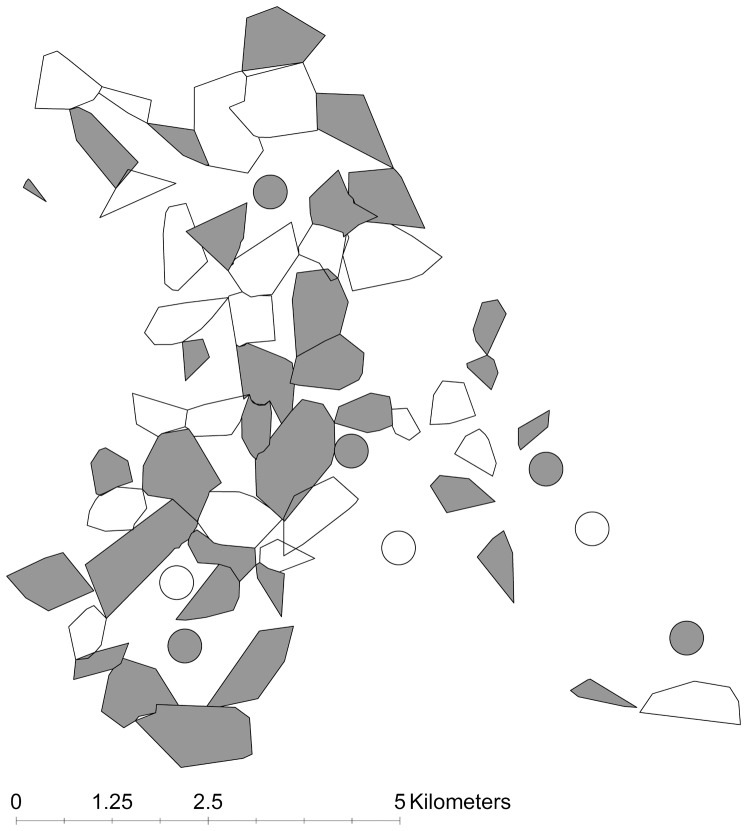
Configuration of badger social group territories in the first year of the study. Territories have been derived from bait marking data and show allocation of vaccine treatment (shaded areas) and control (open areas). Circles indicate additional main setts located after the 2006 bait marking for which the territorial boundaries could not be delineated until the following year, but which were determined to represent discrete social groups in 2006 and allocated a treatment accordingly. Additional badger groups may have been present in non-surveyed areas adjacent to the mapped territories, particularly around the edge of the study area.

Field work was carried out in an area of mixed woodland and agricultural land covering approximately 55 km^2^ in Gloucestershire, southwest England, between November 2005 and October 2009. The area was chosen as it is within a region where very high badger density has been recorded (25.3 badgers/km^2^ [34]), where TB is known to be endemic in the badger population [35] and where there had not been recent badger culling. Badger social group territories were identified by bait marking [36], with individuals being assigned to social groups according to the location of their capture. Social groups were allocated to “vaccinate” or “control” treatment following a baseline trapping session in summer 2006 [Table S1, Figs. 1 and S1]. Treatments were allocated at a ratio of 60∶40 (vaccinate∶control) as this ratio was determined, through modelling known parameters, to have the most statistical power to detect a difference between treatments if one existed (unpublished data). TB may be spatially aggregated in badger populations [35] and badger social groups may vary considerably in size [27]. A stratified randomisation process that accounted for variation in group sizes and prevalence of infection was accordingly used to allocate treatments between social groups (see [24] for full details). Once a social group had been allocated as a vaccinate group, all animals first captured in that group were vaccinated. Animals originating from vaccinate groups but caught in subsequent years in a control group were repeat vaccinated according to their original treatment allocation. To account for any differences in the force of infection between social groups that merged and fragmented during the study and those that were apparently more stable, we derived a ‘super-group’ variable. This label was assigned to clusters of two to three groups that merged with or subsequently split from one another during the study period.

### Badger trapping, sampling and vaccination

Badgers were captured in steel mesh traps baited with peanuts and set for two consecutive nights following a three to ten-day pre-baiting period. Saturation trapping was carried out, whereby more traps were deployed in the immediate vicinity of the sett than the anticipated number of resident badgers. All active setts in the study area were trapped at least twice a year, other than in 2007 when a foot and mouth disease outbreak prevented the second trapping operation from taking place (Table S1). Each badger was anaesthetised by intra-muscular injection of a combination of ketamine hydrochloride (100 mg ml^−1^, Vetalar ™ V, Pharmacia & Upjohn, Crawley, UK), medetomidine hydrochloride (1 mg ml^−1^, Domitor ®, Pfizer, Sandwich, UK) and butorphanol tartrate (10 mg ml^−1^, Torbugesic ®, Fort Dodge Animal Health Ltd, Southampton, UK) at a ratio of 2∶1∶2 by volume respectively [37]. This was supplemented with inhalant isoflurane when necessary. Upon first capture each animal was marked with an identifier microchip (inserted subcutaneously between the shoulders) and a tattoo on the abdomen with the corresponding unique three-digit identification number. For each capture event, the trap location, sex and age class (<1 year = cub, ≥1 year = adult) were recorded. Tooth wear (five categories from none (0%) to worn flat (100%)) was recorded as a proxy for age [38]. Clinical samples were taken from all badgers at each capture event, where possible. These consisted of tracheal mucus (by catheter); urine (by manual expression or catheter); faeces (by Microlax enema); and swabs of any wounds, discharges or abscesses. Blood samples were taken into heparin and SST BD Vacutainer Blood Collection Tubes (BD, Plymouth, UK) and processed on the same day. BCG Danish strain 1331 vaccine (Statens Serum Institut (SSI), Copenhagen, Denmark) was supplied at 2–8×10^6^ colony-forming units (CFU) per vial. The vaccine was prepared by adding 1 ml of Sauton diluent to each vial. The vaccine was injected in the left or right lumbar muscle, following shaving and cleaning of the overlying skin. All animals allocated to vaccinate groups received 1 ml of vaccine that had been reconstituted for less than 4 h. BCG vaccine was administered on recapture at a rate of one dose per calendar year, resulting in some individuals receiving multiple vaccinations over the course of the study. After sampling and treatment, captured badgers were returned to their point of capture and released.

### Diagnostics

We used bacterial culture for *M. bovis* to identify active excretion [39] and the Brock (TB) Stat-Pak test [40] as evidence of more progressed infection for analyses of both a direct and indirect effect of BCG vaccination. We also used the more sensitive IFNγ release assay (IGRA PPDB – PPDA) for our analyses of an indirect effect of vaccination in unvaccinated badgers. For our investigation of the direct effects of vaccination, we substituted the IGRA (PPDB – PPDA) results with those from an alternative, less sensitive, test format based on the use of specific *M. bovis* antigens ESAT-6 and CFP-10, because the performance of the former test may be compromised by BCG vaccination leading to reduced specificity [24], [25]. To be as robust as possible, we adopted a “quadruple test” of infection by combining the outcomes of all four diagnostic tests (PPDB – PPDA, ESAT-6 & CFP-10, Stat-Pak and culture) to identify animals likely to be infected at their time of first capture. The quadruple test was used only as a filtering tool and not as a response variable in any of the following analyses.

Each of the four diagnostic tests had a different sensitivity and specificity for detecting *M. bovis* infection in badgers and we calculated the range in sensitivity of the combined tests using the union of the individual probabilities from published data (Table S2). The sensitivity of the combined triple test*_V_* (positive for one or more of IGRA ESAT-6 & CFP10, Stat-Pak or culture) used in the investigation of a direct effect of vaccination was calculated to lie between 61% and 86% (all badgers). The sensitivity of the triple test*_UV_* (positive for one or more of IGRA PPDB-PPDA, Stat-Pak or culture) used in the investigation of an indirect effect lay between 85% and 94% for adults and between 57% and 84% for cubs (Table S2). Each represented the most sensitive means to detect *M. bovis* infection in *vaccinated* and *unvaccinated* animals, respectively, in the absence of post-mortem examination. Test specificity could not be similarly enhanced by taking this approach but specificity of all tests was relatively high (range 0.93–1.0; Table S2).

### Statistical analyses

#### (i) Investigating the direct effect of vaccination on individual badgers

We investigated the impact of vaccination on the transition from test-negative to test-positive status in individual badgers using event history analysis. For consistency with previous analysis we ran separate analyses for test results in isolation and in combination with each other. The ‘event’ was therefore one of the following: a positive outcome to each diagnostic test in isolation (i.e. IGRA ESAT-6 & CFP-10, Stat-Pak and culture); a positive outcome to Stat-Pak and/or culture when both were considered (“dual test”); or a positive result to at least one of all three tests when considered together (“triple test*_V_*”), at each capture point after the start of vaccination. We developed models that assumed a baseline hazard common to all social groups and used relevant covariates (Table S3) in Cox-proportional hazards models [41]. The risk of an individual being culture-positive has been shown to be related to the presence of other culture-positive (i.e. infectious, actively excreting) animals in its social group [35], [42], therefore a covariate describing group infection status (presence of culture positive badgers) was included (Table S3). It was not possible to model the multiplicative effects of two factors using the above approach. We therefore attempted to model the interaction between group infection status and treatment as four factors (vaccinated/culture positives present, unvaccinated/culture positives present, vaccinated/culture positives absent, unvaccinated/culture positives absent), but there were insufficient cases of unvaccinated badgers captured in groups with culture positive badgers present to model this class.

As individuals were captured and tested at intervals and not all individuals were captured at each trapping session, the data were interval censored; that is, a disease incidence event would have occurred at a point prior to testing and therefore should not be ascribed simply to the sampling point at which it was detected. Interval censoring leads to bias which impacts on both the direction and magnitude of the effects of covariates in the Cox model, the magnitude of which cannot be predicted *a priori*. We therefore used the iterative convex minorant algorithm (ICM) as implemented in the intcox library [43] in the R statistical package (R Development Core Team 2010) to estimate the regression coefficients of the Cox model. Since this procedure cannot estimate standard errors for the regression coefficients, we used a bootstrapping procedure to create 95% confidence intervals for the exponent of each coefficient based on 999 re-samples of the original data. A covariate was considered to be non- significant if the confidence intervals around the bootstrap estimates included 1. The final model was obtained through stepwise removal of non-significant covariates from the full model.

#### (ii) Investigating the indirect effect of vaccination on unvaccinated badgers

Although all animals first captured in vaccinate groups were vaccinated from Autumn 2006 onwards, only a proportion of the group would have been captured at the first, or even subsequent, capture event(s). In addition, there was annual recruitment of cubs into most vaccinate groups, some evidence of previously discrete vaccinate and control groups merging to form a larger vaccinate super-group and, we suspect, immigration of previously uncaptured animals from control groups or groups outside of the study. Consequently, at any given capture event following the start of vaccination the proportion of animals captured that had been previously vaccinated is likely to have been less than one and this proportion varied among groups (Table S4). We used mixed effect models to assess the relationship between *M. bovis* infection status at first capture of unvaccinated individuals from vaccinate social groups and the proportion of all other group members captured at that time that had been vaccinated previously. We used the combined outcomes of IGRA PPDB – PPDA, Stat-Pak and culture (triple test*_UV_*) to infer infection, as this was the most sensitive means to detect *M. bovis* infection in an unvaccinated live animal. Analysis of mixed effect models for non-normal data is problematic because the results of analyses may not be stable and some methods of estimation lead to bias in the estimates of model parameters [44]. We therefore used Laplace approximation [45], [46], following the approach used by Rushton et al. [47], which has the advantage of approximating true GLMM likelihood and is consequently more robust than using penalised quasi-likelihood which can lead to biased parameter estimates if the standard deviation in the random effect is large [44]. We ran analyses for cubs and adults separately due to the potential for pseudo-vertical disease transmission to impede vaccination, and because previous work showed the proportion of infected cubs in a social group was positively related to the presence of infectious adult females [35]. We hypothesised that the force of infection within their group would diminish as an increasing proportion of the residents were vaccinated. There were insufficient data to restrict analysis to proportions/numbers of particular age or sex groups that had been previously vaccinated e.g. breeding females. We expected any observable effect on adults to be weaker than in cubs, due to the propensity for adults to travel further from their resident social group, thus increasing their likelihood of encountering infection. Also, animals captured for the first time as adults may have already been infected with *M. bovis*, prior to other group members being vaccinated.

We included the proportion of contemporary group residents that had been previously vaccinated as a continuous variable in a model including the time point and season when badgers were first captured (i.e. the point at which they entered the study), along with the sex of the individual and the presence of culture-positive animals within their social group as covariates (Table S3). Badger cubs are generally born at the same time of the year [27] so date of first capture represents a crude proxy for age at first capture. We also ran analyses using several categorical classifications (including the same covariates) whereby the proportion of animals previously vaccinated was assigned into classes e.g. 0–0.33 etc. for illustrative purposes to aid the interpretation of the more rigorous continuous model results. In view of the arbitrary nature of categorisation we investigated a range of cut-offs, although finer scale categorisation was limited by sample sizes. Only individuals caught for the first time during or after the second year of the study were considered for analysis as this was the first opportunity for animals to have indirectly benefited from the initial vaccination of other group members. Social group was included as a random factor to allow for unmeasured variation associated with repeated sampling of the same groups. All analyses were undertaken in the R statistical package (R Development Core Team 2010) using the glmmML libraries [45].

## Results

A total of 793 individual badgers were captured on or after the second trap event when BCG was first administered. Of these, 400 were captured more than once, making them potentially eligible for the investigation of the direct effect of vaccination. The remaining 393 were only eligible for the analysis of an indirect (herd immunity) effect of vaccination. The number of social groups varied from year to year due to the recruitment of new groups over time and, to a lesser extent, the dynamic nature of a handful of groups, resulting in an increase from 62 to 85 discrete social groups over the course of the study (Figs. 1 and S1). This included the formation of eight super-groups and six social groups that were deemed to have split into two discrete groups in at least one year of the study. Simple prevalence estimates indicated a reduction in the population-level prevalence of infection (using the combined results from IGRA ESAT-6 & CFP-10, Stat-Pak and culture) over the lifetime of the study (Table S5). Prevalence for the population as a whole based on the combined outcomes of the above three tests reduced from 53% in 2006 to 35% in 2009. Reductions in prevalence were observed in both vaccinate and control groups (Table S5).

### Direct effect of BCG vaccination on individual disease risk

A total of 252 individuals qualified for the event history analysis, following the removal of those individuals caught only once and those that were likely to have been infected prior to vaccination. Vaccination reduced the likelihood of developing a positive test result (after initially testing negative to all tests) by 76% (Odds ratio = 0.24, 95% confidence interval (CI) 0.11–0.52; Table 1) and 54% (Odds ratio = 0.46, 95% CI 0.26–0.88; Table 1), respectively, using the dual and triple diagnostic tests for *M. bovis*. There was no detectable effect of vaccination on the risk of a positive test result for culture alone (data not shown). Badgers were significantly more likely to test positive if there was at least one other culture-positive badger captured in its social group (all tests; Table 1). Male badgers were three times (Odds ratio = 3.01, 95% CI 1.44–5.55) more likely to become positive for one or more of the diagnostic tests than females (triple test*_V_*; Table 1). There was no detectable effect on risk of an individual developing a positive result to any test associated with age, social group membership or the number of individuals captured in its social group (data not shown).

**Table 1:** Effects of BCG vaccination on the risk of individual badgers testing positive to a suite of diagnostic tests for *M. bovis*.

Diagnostic test	factor	odds ratio^a^	SD^b^	lower 95% CI	upper 95% CI
IGRA (ESAT-6/CFP-10)	Presence of culture-positive badgers	6.43	2.24	3.25	12.53
	Vaccinated previously	0.42	0.14	0.23	0.84
	Sex (male)	2.44	0.89	1.19	4.30
Stat-Pak	Presence of culture-positive badgers	4.85	2.68	1.87	10.88
	Vaccinated previously	0.15	0.22	0.06	0.42
Culture	Presence of culture-positive badgers	3.99	1.54	1.77	7.65
Stat-Pak or culture (dual test)	Presence of culture-positive badgers	7.92	3.37	3.42	16.37
	Vaccinated previously	0.24	0.43	0.11	0.52
Triple test*_V_* c	Presence of culture-positive badgers	6.20	2.33	3.14	11.48
	Vaccinated previously	0.46	0.12	0.26	0.88
	Sex (male)	3.01	1.44	1.44	5.55

Outcome of the final event analysis model showing the individual risk of testing positive for each of the diagnostic tests and test combinations, SD and 95% confidence intervals associated with different explanatory factors. Only significant factors are listed.

a Odds ratios are equivalent to the mean exponent of the coefficient (exp(ß)) and represent change in odds associated with an individual badger testing positive for *M. bovis* in relation to the relevant covariate being assessed. Odds ratio <1 = decreased odds (negative ß coef.); >1 = increased odds (positive ß coef.).

b Standard deviation of the coefficient.

c Triple test*_V_* is positive for one or more of IGRA (ESAT-6/CFP-10), Stat-Pak, or culture.

### Indirect effect of BCG vaccination on disease risk in unvaccinated individuals

In total, 121 adults and 185 cubs (all unvaccinated at the time of capture) were included in the analysis of an indirect effect of vaccination. Of these, 42 (34.7%) adults and 39 cubs (21.1%) tested positive to one of the three diagnostic tests at first capture. This compared to a prevalence of 51.6% (adults) and 32.8% (cubs) in control groups over the same time period. The probability of an unvaccinated cub from a vaccinate group testing positive to *M. bovis* was significantly inversely related to the proportion of badgers trapped in the same social group at the same capture event that had previously been vaccinated (Odds ratio = 0.08, 95% CI 0.01–0.76; *P* = 0.03; Table 2). However, the probability of an unvaccinated adult testing positive to *M. bovis* was not significantly related to the proportion of group members captured concurrently that had been previously vaccinated (Odds ratio = 0.59, 95% CI 0.06–5.59; *P* = 0.64).

**Table 2:** Factors affecting the likelihood of unvaccinated badgers in vaccinate groups testing positive to any of the following diagnostic tests: IGRA (PPDB – PPDA); Stat-Pak; culture.

	odds ratio^a^	SE^b^	lower 95% CI	upper 95% CI	*z*-value^c^	*p*
**Cubs**						
Intercept^d^	0.26	0.50	0.10	0.68	−2.73	0.006
Presence of culture-positive badgers	3.86	0.53	1.37	10.90	2.55	0.01
Proportion of group previously vaccinated^e^	0.08	1.17	0.01	0.76	−2.19	0.03
**Adults**						
Intercept	0.13	0.53	0.05	0.37	−3.84	<0.001
Presence of culture-positive badgers	6.50	0.60	1.99	21.24	3.11	0.002
Sex (male)	3.70	0.53	1.30	10.53	2.46	0.01

The proportion of badgers previously vaccinated is modelled as a continuous variable. Badger social group was fitted as a random factor and only significant factors are listed.

a Odds ratios are equal to the mean exponent of the coefficient (exp(ß)) and represent change in the odds associated with an individual badger testing positive for *M. bovis* in relation to the relevant covariate being assessed. Odds ratio <1 = decreased odds (negative *z*-value); >1 = increased odds (positive *z*-value).

b Standard error of the coefficient.

c Coefficient divided by the SE of the coefficient.

d The intercept represents the odds of testing positive for *M. bovis* for an individual badger in a social group without culture-positive individuals and where no other group members have been vaccinated.

e The number of other previously vaccinated badgers divided by the total number of other badgers caught in a social group at the time that an unvaccinated badger was first caught and tested.

**Figure 2:**
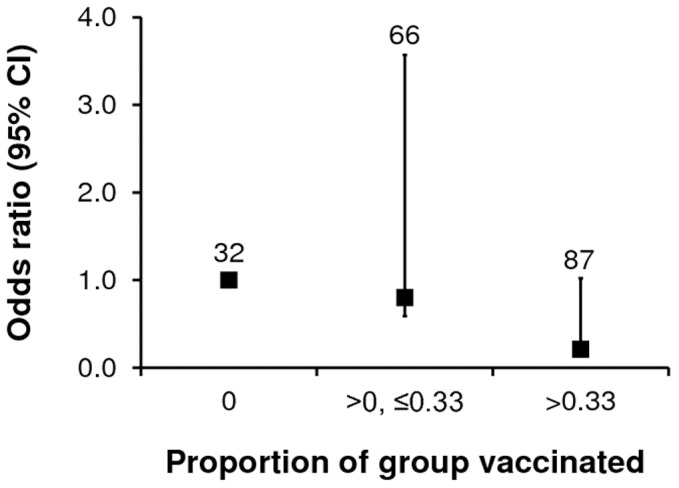
Decreasing risk of an unvaccinated badger cub testing positive to a triple diagnostic test for *M. bovis* infection as the proportion of vaccinated badgers in its social group increases. The number of cubs within each category is shown.

When we assigned the proportion of previously vaccinated animals to discrete categories, there was a consistent trend for the highest levels of vaccination to show the greatest reduction in risk to unvaccinated cubs (Table S6) The reduced risk of an unvaccinated cub yielding a positive test result ranged between 79% and 86% for the highest levels of vaccination in the three categorical models, although this only approached statistical significance in one of the models (Table S6). For example, the probability of an unvaccinated cub testing positive to *M. bovis* was reduced by 79% when more than one third of the contemporary group residents had been vaccinated previously (Odds ratio = 0.21, 95% CI 0.05–0.81; *P* = 0.02; Fig. 2).

There was a strong positive association between unvaccinated cubs and adults that tested positive for *M. bovis*, and the presence at capture of culture-positive, i.e. *M. bovis*-excreting, animals in the same social group (Table 2). Adult male badgers were almost four times more likely to yield a positive result than adult females (Odds ratio = 3.7, 95% CI 1.30–10.53; *P* = 0.01; Table 2). Interactions between significant main effects in the model were investigated but none were found to be significant (data not shown).

## Discussion

Vaccination was associated with a significantly lower risk of an individual badger testing positive to both a triple diagnostic test for TB infection and a dual diagnostic test as a proxy for more advanced infection. In the absence of associated *post-mortem* data, tests based on measurement of an immune response are not proof positive of TB infection. Similarly, the absence of a positive test result does not mean the individual is certainly free of infection. However, the triple test*_V_* used here is the most sensitive and specific measure of *M. bovis* infection in a live vaccinated badger and so provides confidence that these results are biologically meaningful.

The effect of vaccination on the triple test*_V_* outcome was to reduce the risk of a positive result by 54% in vaccinated individuals. Without *post-mortem* data it was not possible to ascertain what proportion of the triple test*_V_*-negative, vaccinated badgers were protected from infection and what proportion still acquired infection, but were not detected using the triple test*_V_*. It is unsafe to assume that triple test*_V_* negativity equates to the absence of infection. A greater estimate of vaccine effect (76%) was observed with the dual test. The IGRA (ESAT-6/CFP-10) was absent from the dual test. As the IGRA is more sensitive than either the Stat-Pak or culture at detecting *M. bovis* infection in live badgers, this result was not entirely unexpected. Given that badgers are less likely to be dual test-positive if they are at an earlier stage of the disease process [48], this result is consistent with an additional impact of vaccination on the prevention of disease progression in those vaccinated animals that still succumbed to infection.

In addition to demonstrating a significant direct benefit of BCG vaccination in individual badgers, we show a significant indirect effect of vaccination on unvaccinated cubs born into vaccinated groups. We conclude from these findings that unvaccinated, susceptible badger cubs were indirectly protected from disease transmission through a “herd immunity” effect, at least up to the point at which they were above ground and could be trapped and vaccinated. Whilst we cannot categorically rule out other potential mechanisms for this result, it is unlikely that unvaccinated cubs were afforded direct protection from maternal transfer of antibodies arising from BCG vaccination of the mother (passive immunity) as there is no evidence of a significant protective effect in cubs from maternal antibody transfer in naturally infected badger populations [49], where the antigen load would be expected to be higher than following vaccination. The probability of BCG being transferred from mother to cub via suckling or across the placenta (vertical transmission) is extremely low as there is no evidence of excretion and minimal evidence of dissemination of BCG in vaccinated individuals [50]. However, even in the unlikely event that some unvaccinated cubs were afforded protection directly from their mothers through either of the above mechanisms, the practical implications of these results remain unchanged. The failure to detect a significant herd effect in adults may be partly explained by differences in their ranging behaviour. Adults are more likely to range further than cubs, leading to a higher risk of encountering direct or indirect sources of infection outside of their usual social environment, for example from badgers or their latrines in unvaccinated control groups, neighbouring groups of unknown infection status or from other species. A contributing factor to the observed difference is that animals first captured as adults in a vaccinated group may not have been resident in that group and/or may have already been infected prior to the start of BCG vaccination.

The crude categorisation of the proportion of vaccinated badgers into discrete classes did not permit the identification of a “vaccination threshold” beyond which a significant herd effect in cubs is likely to occur, but these results clearly demonstrate an increasing indirect benefit of vaccination to susceptible badger cubs as an increasing proportion of their social group is vaccinated.

The presence of infectious individuals within a social group represents a high risk of infection for susceptible residents. An individual badger was on average nearly eight times more likely to yield a positive dual-test result (associated with increased disease severity or progression) where at least one other badger captured in its social group was found to be excreting *M. bovis*, than if no other badgers in the group were found to be excreting at the time of capture. This result is not surprising given the high degree of sociality and territoriality among badgers and is consistent with findings from a long-term field study of *M. bovis* infection in badgers which showed the proportion of culture-positive animals in a group to be the most significant factor influencing the risk of other individuals becoming culture positive within any given year [35], [42]. From a disease management perspective, it is noteworthy that the significant reductions in infection risk to individual badgers as a result of vaccination were apparent, even in the presence of individuals with evidence of advanced infection within the social group and within a relatively short period of time since the start of vaccination.

Vaccination of badgers with BCG appears to be beneficial in at least two ways: by directly reducing the TB burden in vaccinated individuals [24], [25] and by indirectly reducing the risk of unvaccinated cubs acquiring infection, most likely through a herd immunity effect on this susceptible component of the badger population. Indirect ‘protection’ bestowed upon juveniles before they become accessible for vaccination themselves could be an important contribution to the success of vaccinating wildlife. Heterogeneity in contact and transmission rates among human communities influences the magnitude of herd immunity and in turn its contribution to the success of mass immunisation programmes [31]. The stable social structure of badgers and relatively limited contact between groups [51] has previously been shown to impede disease spread [35], [42]. The marked difference in the indirect impact of vaccination on adults and cubs observed in the present study indicates another way in which the social organisation of badger populations may influence disease transmission among age classes within the social group, although we might expect to see a similarly protective effect in adults over a longer time period as the benefits of herd immunity accrue in the population. Contact patterns among other wild animals are likely to be equally important in determining the impact of vaccination in controlling disease. These results also emphasise the importance of considering indirect as well as direct measures of vaccine efficacy when evaluating vaccination as a strategy for wildlife disease control.

## Conclusion

Vaccinating free-living wild badgers with BCG significantly reduced the risk of an individual developing a positive result to a range of diagnostic tests used as a proxy for *M. bovis* infection. In particular, an additional protective effect was observed using a dual test associated with more advanced/severe disease. Moreover, we have demonstrated an indirect benefit of vaccination for susceptible, unvaccinated badger cubs before they became available for vaccination themselves. Together, these results provide additional insights into the nature of the protective effect of BCG vaccination of wild badgers in their natural social setting. Our findings should be considered in light of the relatively short time scale over which the beneficial effects of vaccination were observed.

## Supporting Information

Figure S1 **Configuration of badger social group territories between 2006 and 2009.** Allocation of vaccine treatment (shaded areas) and control (open areas) is shown. Circles indicate additional main setts located after bait marking of that year and for which the territorial boundaries could not be delineated until the following year, but which were determined to represent discrete social groups and allocated a treatment accordingly. New groups were recruited in some years and a minority of groups merged together to form a “super-group” and/or split apart to form two smaller groups over the course of the study. Additional badger groups may have been present in non-surveyed areas adjacent to the mapped territories, particularly around the edge of the study area.
(DOC)

Table S1 **Number of badgers captured at each successive capture event during the four-year field study, together with approximate capture dates.**
(DOC)

Table S2 **Estimated sensitivities and specificities for each diagnostic test and test combinations used in the analyses.**
(DOC)

Table S3 **Covariates included in the investigation of the direct^D^ and indirect^I^ effects of BCG vaccination.**
(DOC)

Table S4 **The mean proportion of badgers, captured in vaccinate groups at each successive capture event, that had been vaccinated previously.** The proportion of all badgers previously vaccinated (A) and previously vaccinated adults only (B) are shown, together with Standard deviation and the number of social groups from which badgers were captured. Capture events prior to or at the start of vaccination (T1–T3) are not listed.
(DOC)

Table S5 **Simple TB prevalence estimates for the study population over the four years of the study.** Estimates are based on the first test result of all badgers captured each year throughout the whole study population (A), groups receiving the vaccinate treatment (B) and experimental control groups where no vaccine was administered (C). Prevalence estimates are provided for each of the diagnostic tests that are appropriate for use on a vaccinated population. Prevalence estimates for Stat-Pak and culture when considered together (dual test) are shown together with those when the results of all three diagnostic tests are considered (triple test*_v_*). Prevalence is derived from the proportion of animals testing positive (n) over the total number with a valid test result (N).
(DOC)

Table S6 **Factors affecting the likelihood of unvaccinated badger cubs in vaccinate groups testing positive to any of the following diagnostic tests: IGRA (PPDB – PPDA); Stat-Pak; culture, where the proportion of badgers previously vaccinated is modelled as a range of categorical variables (a–c).**
(DOC)
