# Supplementary material for: BCG Vaccination Reduces Risk of Tuberculosis Infection in Vaccinated Badgers and Unvaccinated Badger Cubs
Source: PLoS One. 2012 Dec 12;7(12):e49833. doi: 10.1371/journal.pone.0049833 (PMC3521029; doi:10.1371/journal.pone.0049833)
Supplement: Figure S1 — Configuration of badger social group territories between 2006 and 2009. Allocation of vaccine treatment (shaded areas) and control (open areas) is shown. Circles indicate additional main setts located after bait marking of that year and for which the territorial boundaries could not be delineated until the following year, but which were determined to represent discrete social groups and allocated a treatment accordingly. New groups were recruited in some years and a minority of groups merged together to form a “super-group” and/or split apart to form two smaller groups over the course of the study. Additional badger groups may have been present in non-surveyed areas adjacent to the mapped territories, particularly around the edge of the study area. (DOC) [file pone.0049833.s001.doc]

**Figure S1. Configuration of badger social group territories between 2006 and 2009.**

Allocation of vaccine treatment (shaded areas) and control (open areas) is shown. Circles indicate additional main setts located after bait marking of that year and for which the territorial boundaries could not be delineated until the following year, but which were determined to represent discrete social groups and allocated a treatment accordingly. New groups were recruited in some years and a minority of groups merged together to form a “super-group” and/or split apart to form two smaller groups over the course of the study. Additional badger groups may have been present in non-surveyed areas adjacent to the mapped territories, particularly around the edge of the study area.
