# Supplementary material for: BCG Vaccination Reduces Risk of Tuberculosis Infection in Vaccinated Badgers and Unvaccinated Badger Cubs
Source: PLoS One. 2012 Dec 12;7(12):e49833. doi: 10.1371/journal.pone.0049833 (PMC3521029; doi:10.1371/journal.pone.0049833)
Supplement: Table S1 — Number of badgers captured at each successive capture event during the four-year field study, together with approximate capture dates. (DOC) [file pone.0049833.s002.doc]

| **Capture event** | **Date captured** | **Number of badgers caught** |
| --- | --- | --- |
| T1*^a^* | June/July 2006 | 257 |
| T2 | Sept./Oct. 2006 | 182 |
| T3*^b^* | Sept./Oct./Nov. 2006 | 135 |
| T4 | June/July 2007 | 311 |
| T5*^c^* | Cancelled | - |
| T6 | June/July 2008 | 219 |
| T7 | Sept./Oct. 2008 | 176 |
| T8 | June/July 2009 | 276 |
| T9 | Sept./Oct. 2009 | 231 |

**Table S1. Number of badgers captured at each successive capture event during the four-year field study, together with approximate capture dates.**

*^a^*Initial (base-line) trapping session to estimate social group size and group disease prevalence (based on positive IFNγ release assay (IGRA), PPD-B – PPD-A results) in order to allocate treatment (vaccinate, control). BCG vaccine was first administered to animals captured within vaccinate groups at T2. *^b^*Social groups trapped two weeks after T2 to collect vaccine safety data (not reported here). *^c^*Autumn trapping session cancelled due to an outbreak of Foot and Mouth Disease.
