# Supplementary material for: BCG Vaccination Reduces Risk of Tuberculosis Infection in Vaccinated Badgers and Unvaccinated Badger Cubs
Source: PLoS One. 2012 Dec 12;7(12):e49833. doi: 10.1371/journal.pone.0049833 (PMC3521029; doi:10.1371/journal.pone.0049833)
Supplement: Table S2 — Estimated sensitivities and specificities for each diagnostic test and test combinations used in the analyses. (DOC) [file pone.0049833.s003.doc]

**Table S2. Estimated sensitivities and specificities for each diagnostic test and test combinations used in the analyses.**

| **diagnostic test** | **estimated test sensitivity** | **estimated test specificity** | **reference** |
| --- | --- | --- | --- |
| IGRA, PPDB-PPDA | 0.85 (adults) | 0.93 (adults) | Chambers et al. 2009 |
|  | 0.57 (cubs) | 0.98 (cubs) | Chambers et al. 2009 |
| IGRA, ESAT-6/CFP-10 | 0.61 | 0.95 | Dalley et al. 2008 |
| Stat-Pak | 0.54 | 0.93 | Chambers et al. 2009 |
| Culture | 0.20 | 1.00 | Chambers et al. 2011 |
| Stat-Pak or culture | 0.54 - 0.63*^c^* | - | - |
| Triple test*^a^* (incl. IGRA, ESAT-6/CFP-10) | 0.61 - 0.86*^d^* | - | - |
| Triple test*^b^* (incl. IGRA, PPDB-PPDA) | 0.85 - 0.94*^d^* (adults) | - | - |
|  | 0.57 - 0.84*^d^* (cubs) | - | - |

*^a^*Positive for one or more of IGRA (ESAT-6/CFP-10), Stat-Pak, or culture. *^b^*Positive for one or more of IGRA (PPDB-PPDA), Stat-Pak, or culture. *^c^*P(Stat-Pak ∪ Culture) = P(Stat-Pak) + P(Culture) – P(Stat-Pak ∩ Culture). *^d^*P(IGRA ∪ Stat-Pak ∪ Culture) = P(IGRA) + P(Stat-Pak) + P(Culture) – P(IGRA ∩ Stat-Pak) – P(Stat-Pak ∩ Culture) – P(IGRA ∩ Culture) + P(IGRA ∩ Stat-Pak ∩ Culture).
