# Supplementary material for: BCG Vaccination Reduces Risk of Tuberculosis Infection in Vaccinated Badgers and Unvaccinated Badger Cubs
Source: PLoS One. 2012 Dec 12;7(12):e49833. doi: 10.1371/journal.pone.0049833 (PMC3521029; doi:10.1371/journal.pone.0049833)
Supplement: Table S3 — Covariates included in the investigation of the directD and indirectI effects of BCG vaccination. (DOC) [file pone.0049833.s004.doc]

| **covariates** | **description** | **values (data type)** |
| --- | --- | --- |
| Tooth-wear^D^ | Estimated category of tooth-wear as a surrogate for age in years | 0 per cent; 25 per cent; 50 per cent; 75 per cent; 100 per cent (categorical) |
| Sex^D, I^ |  | male; female (categorical) |
| Super-group member^D^ | Is the individual at the current time step a member of a super-group? | yes; no (binary) |
| Vaccinated^D^ | Has the individual been vaccinated previously? | yes; no (binary) |
| Multiple vaccination^D^ | How often has the individual been vaccinated previously? | never; once; repeatedly (categorical) |
| Presence of culture-positive badgers^D, I^ | Are there any culture positive members in the social group at the current time step? | yes; no (binary) |
| Proportion of group vaccinated^I^ | A factor indicating the proportion of the group members trapped at this time point that had been vaccinated previously | continuous |
| Group size ^I^ | Number of members of the social group captured at the current time step as a proxy for absolute group size | integer (number) |
| Capture event^I^ | Time point (T1, T2 etc.) at which the individual was first captured | integer (number) |
| Season^I^ | Season in which the individual was first captured | summer; autumn (categorical) |

**Table S3. Covariates included in the investigation of the direct^D^ and indirect^I^ effects of BCG vaccination.**
