# Supplementary material for: BCG Vaccination Reduces Risk of Tuberculosis Infection in Vaccinated Badgers and Unvaccinated Badger Cubs
Source: PLoS One. 2012 Dec 12;7(12):e49833. doi: 10.1371/journal.pone.0049833 (PMC3521029; doi:10.1371/journal.pone.0049833)
Supplement: Table S4 — The mean proportion of badgers, captured in vaccinate groups at each successive capture event, that had been vaccinated previously. The proportion of all badgers previously vaccinated (A) and previously vaccinated adults only (B) are shown, together with Standard deviation and the number of social groups from which badgers were captured. Capture events prior to or at the start of vaccination (T1–T3) are not listed. (DOC) [file pone.0049833.s005.doc]

| **Table S4. The mean proportion of badgers, captured in vaccinate groups at each successive capture event, that had been vaccinated previously.** The proportion of all badgers previously vaccinated (A) and previously vaccinated adults only (B) are shown, together with Standard deviation and the number of social groups from which badgers were captured. Capture events prior to or at the start of vaccination (T1-T3) are not listed. | | | | | | |
| --- | --- | --- | --- | --- | --- | --- |
| **(A) All badgers (adults and cubs)** | | | | | | |
| Capture event | summer 2007 (T4) | autumn 2007*^a^* (T5) | summer 2008 (T6) | autumn 2008 (T7) | summer 2009 (T8) | autumn 2009 (T9) |
| Proportion vaccinated | 0.31 | No data | 0.46 | 0.73 | 0.54 | 0.77 |
| SD | 0.36 | No data | 0.38 | 0.35 | 0.32 | 0.32 |
| Number of groups | 41 | No data | 44 | 38 | 45 | 44 |
| **(B) Adults only** | | | | | | |
|  | summer 2007 (T4) | autumn 2007 (T5) | summer 2008 (T6) | autumn 2008 (T7) | summer 2009 (T8) | autumn 2009 (T9) |
| Proportion vaccinated | 0.48 | No data | 0.54 | 0.78 | 0.70 | 0.94 |
| SD | 0.32 | No data | 0.31 | 0.32 | 0.32 | 0.32 |
| Number of groups | 39 | No data | 42 | 34 | 44 | 39 |

Note general trend for the vaccinated proportion to increase over time, with a tendency for the proportion vaccinated to decrease slightly in summer. This is partly due to the recruitment of non-vaccinated cubs into the population at this time, although a similar pattern is observed when only the proportion of previously vaccinated adults is considered (B). *^a^*Autumn 2007 trapping session cancelled due to an outbreak of Foot and Mouth Disease.
