# Supplementary material for: BCG Vaccination Reduces Risk of Tuberculosis Infection in Vaccinated Badgers and Unvaccinated Badger Cubs
Source: PLoS One. 2012 Dec 12;7(12):e49833. doi: 10.1371/journal.pone.0049833 (PMC3521029; doi:10.1371/journal.pone.0049833)
Supplement: Table S5 — Simple TB prevalence estimates for the study population over the four years of the study. Estimates are based on the first test result of all badgers captured each year throughout the whole study population (A), groups receiving the vaccinate treatment (B) and experimental control groups where no vaccine was administered (C). Prevalence estimates are provided for each of the diagnostic tests that are appropriate for use on a vaccinated population. Prevalence estimates for Stat-Pak and culture when considered together (dual test) are shown together with those when the results of all three diagnostic tests are considered (triple testv). Prevalence is derived from the proportion of animals testing positive (n) over the total number with a valid test result (N). (DOC) [file pone.0049833.s006.doc]

| **Table S5. Simple TB prevalence estimates for the study population over the four years of the study.** Estimates are based on the first test result of all badgers captured each year throughout the whole study population (A), groups receiving the vaccinate treatment (B) and experimental control groups where no vaccine was administered (C). Prevalence estimates are provided for each of the diagnostic tests that are appropriate for use on a vaccinated population. Prevalence estimates for Stat-Pak and culture when considered together (dual test) are shown together with those when the results of all three diagnostic tests are considered (triple test*_v_*). Prevalence is derived from the proportion of animals testing positive (n) over the total number with a valid test result (N). | | | | | | | | | | |
| --- | --- | --- | --- | --- | --- | --- | --- | --- | --- | --- |
| Test | IGRA (ESAT6-CFP10) | | Stat-Pak | | Culture | | Dual test*^a^* | | Triple test*_v_* | |
|  | N | n (% prev.) | N | n (% prev.) | N | n (% prev.) | N | n (% prev.) | N | n (% prev.) |
| **(A) Study population** | | | | | | | | | | |
| 2006 | 344 | 134 (38.95) | 357 | 59 (16.53) | 283 | 33 (11.66) | 283 | 73 (25.80) | 294 | 156 (53.06) |
| 2007 | 300 | 67 (22.33) | 310 | 39 (12.58) | 291 | 17 (5.84) | 295 | 46 (15.59) | 288 | 80 (27.78) |
| 2008 | 296 | 82 (27.70) | 296 | 42 (14.19) | 278 | 24 (8.63) | 278 | 53 (19.06) | 277 | 94 (33.94) |
| 2009 | 365 | 99 (27.12) | 374 | 68 (18.18) | 342 | 30 (8.77) | 347 | 74 (21.33) | 342 | 119 (34.80) |
| **(B) Vaccinates*^a^*** | | | | | | | | | | |
| 2006 | 208 | 86 (41.35) | 217 | 35 (16.13) | 172 | 21 (12.21) | 172 | 44 (25.58) | 182 | 98 (53.85) |
| 2007 | 188 | 38 (20.21) | 195 | 21 (10.77) | 184 | 11 (5.98) | 186 | 27 (14.52) | 181 | 50 (27.62) |
| 2008 | 192 | 45 (23.44) | 192 | 25 (13.02) | 178 | 12 (6.74) | 179 | 31 (17.32) | 180 | 55 (30.56) |
| 2009 | 244 | 67 (27.46) | 245 | 43 (17.55) | 223 | 16 (7.17) | 227 | 45 (19.82) | 228 | 77 (33.77) |
| **(C) Controls** | | | | | | | | | | |
| 2006 | 136 | 48 (35.29) | 140 | 24 (17.14) | 111 | 12 (10.81) | 111 | 29 (26.13) | 112 | 58 (51.79) |
| 2007 | 112 | 29 (25.89) | 115 | 18 (15.65) | 107 | 6 (5.61) | 109 | 19 (17.43) | 107 | 30 (28.04) |
| 2008 | 104 | 37 (35.58) | 104 | 17 (16.35) | 100 | 12 (12.00) | 99 | 22 (22.22) | 97 | 39 (40.21) |
| 2009 | 121 | 32 (26.45) | 129 | 25 (19.38) | 119 | 14 (11.76) | 120 | 29 (24.17) | 114 | 42 (36.84) |

*^a^*Includes vaccinate ‘super-groups’ i.e. previously discrete social groups (vaccinate/vaccinate or vaccinate/control) which were deemed to have merged to form a single group. In addition to recruitment and immigration, new vaccinate and control social groups were recruited into the study each year and a moderate to high proportion of individuals in both treatment groups were considered to be infected at the point of vaccination. Consequently, “vaccinates” includes the test results of a considerable number of individuals that did not benefit from vaccination in the preceding year.
