# Supplementary material for: BCG Vaccination Reduces Risk of Tuberculosis Infection in Vaccinated Badgers and Unvaccinated Badger Cubs
Source: PLoS One. 2012 Dec 12;7(12):e49833. doi: 10.1371/journal.pone.0049833 (PMC3521029; doi:10.1371/journal.pone.0049833)
Supplement: Table S6 — Factors affecting the likelihood of unvaccinated badger cubs in vaccinate groups testing positive to any of the following diagnostic tests: IGRA (PPDB – PPDA); Stat-Pak; culture, where the proportion of badgers previously vaccinated is modelled as a range of categorical variables (a–c). (DOC) [file pone.0049833.s007.doc]

**Table S6. Factors affecting the likelihood of unvaccinated badger cubs in vaccinate groups testing positive to any of the following diagnostic tests: IGRA (PPDB – PPDA); Stat-Pak; culture, where the proportion of badgers previously vaccinated is modelled as a range of categorical variables (a-c).**

|  | odds ratio*^a^* | SE*^b^* | lower 95% CI | upper 95% CI | z-value*^c^* | *p* |
| --- | --- | --- | --- | --- | --- | --- |
| **a)**  Intercept*^d^* | 0.25 | 0.59 | 0.08 | 0.78 | -2.39 | 0.02 |
| Presence of culture-positive badgers | 3.86 | 0.53 | 1.38 | 10.83 | 2.57 | 0.01 |
| Proportion of group previously vaccinated*^e^* | | | | | | |
| >0, ≤0.33 | 0.76 | 0.66 | 0.21 | 2.77 | -0.42 | 0.67 |
| >0.33 | 0.21 | 0.69 | 0.05 | 0.81 | -2.26 | 0.02 |
| **b)**  Intercept | 0.24 | 0.60 | 0.07 | 0.79 | -2.35 | 0.02 |
| Presence of culture-positive badgers | 3.84 | 0.54 | 1.33 | 11.07 | 2.50 | 0.01 |
| Proportion of group previously vaccinated | | | | | | |
| >0, <0.3 | 0.77 | 0.79 | 0.16 | 3.62 | -0.34 | 0.74 |
| ≥0.3, <0.6 | 0.35 | 0.67 | 0.09 | 1.32 | -1.55 | 0.12 |
| ≥0.6 | 0.17 | 0.92 | 0.03 | 1.04 | -1.92 | 0.05 |
| **c)**  Intercept | 0.23 | 0.62 | 0.07 | 0.79 | -2.35 | 0.02 |
| Presence of culture-positive badgers | 4.19 | 0.56 | 1.38 | 12.67 | 2.53 | 0.01 |
| Proportion of group previously vaccinated | | | | | | |
| >0, <0.25 | 0.55 | 0.84 | 0.10 | 3.00 | -0.68 | 0.49 |
| ≥0.25, <0.5 | 0.54 | 0.66 | 0.14 | 1.98 | -0.93 | 0.35 |
| ≥0.5 | 0.14 | 0.84 | 0.03 | 0.75 | -2.30 | 0.02 |

Badger social group was fitted as a random factor. *^a^*Odds ratios are equal to the mean exponent of the coefficient (exp(ß)) and represent change in the odds associated with an individual badger cub testing positive for *M*. *bovis* in relation to the relevant covariate being assessed. Odds ratio < 1 = decreased odds (negative *z*-value); > 1 = increased odds (positive *z*-value). *^b^*Standard error of the coefficient. *^c^*Coefficient divided by the SE of the coefficient. *^d^*The intercept represents the odds of testing positive for *M*. *bovis* for an individual badger cub in a social group without culture-positive individuals and where no other group members have been vaccinated. *^e^*The number of other previously vaccinated badgers divided by the total number of other badgers caught in a social group at the time that an unvaccinated badger was first caught and tested.
